# A cross-sectional study of pandemic influenza health literacy and the effect of a public health campaign

**DOI:** 10.1186/1756-0500-5-377

**Published:** 2012-07-26

**Authors:** Namrata Devi Jhummon-Mahadnac, Jonathan Knott, Caroline Marshall

**Affiliations:** 1 University of Melbourne, Melbourne, VIC, 3050, Australia; 2 Emergency Department, Royal Melbourne Hospital and Department of Medical Education, University of Melbourne, Grattan St, Parkville, VIC, 3050, Australia; 3 Victorian Infectious Diseases Service, Royal Melbourne Hospital and Department of Medicine, University of Melbourne, Grattan St, Parkville, VIC, 3050, Australia

## Abstract

**Background:**

To ascertain the understanding of 2009 pandemic (H1N1) influenza and relevant infection control measures in an emergency department population and to assess the effectiveness of education campaigns in informing the public about the pandemic.

**Methods:**

Questionnaires were administered to patients, visitors, non-clinical staff and volunteers. Data were collected on knowledge, preventative measures, information sources, attitudes to government and media reporting, perceived seriousness, behaviour change and intended compliance with future measures. Results were used to construct an overall knowledge score.

**Results:**

There were 252 participants. Traditional forms of mass media (138 [55%]) remained the principal information source. Approximately 70% (176) accurately described mode of transmission and recommended precautions and 68% (175) reported behaviour change because of the pandemic. Gaps in knowledge included failure to identify certain high risk groups. Recall of government campaigns was significantly associated with a higher knowledge score. 60% (151) thought that authorities and media had exaggerated the threat; only 40% (101) would comply with recommended measures in a future pandemic.

**Conclusions:**

The knowledge regarding pandemic influenza was high in this population and positively affected by official campaigns. Pandemic planning should address knowledge gaps and the impression that authorities had exaggerated the public-health threat.

## Background

Pandemic (H1N1) 2009 influenza represents the first influenza pandemic threat of the 21^st^ century and within eight weeks, all major continents were affected [1]. The virus was initially given the title of “swine flu” although it was subsequently found not to be primarily of swine origin [2]. In Australia, pandemic (H1N1) 2009 influenza was first reported in April 2009.

The pandemic required implementation of the Australian Health Management Plan for Pandemic Influenza (AHMPPI) for the first time after its approval in 2008 [3]. In Victoria, which had the highest notification rates outside of the USA at the outset, numerous prevention campaigns were launched. Information was delivered to health professionals and the public via radio, written press and television promotions as well as on the federal government pandemic website, the Victorian Department of Human Services (DHS) website, twitter feeds and via specific information for indigenous and culturally and linguistically diverse groups.

Whilst other campaigns targeting obesity and substance abuse have been analysed extensively, independent assessment of public health campaigns for pandemic prevention and control is scarce [4]. The success of these campaigns depends on the health literacy of the public about the topic and the perceived susceptibility to the infection or condition. Beliefs about the competency of the authorities and the media in dealing with pandemic information also contribute to understanding [5].

We sought to identify the health literacy of an emergency department (ED) population regarding pandemic (H1N1) 2009 influenza after the peak of the pandemic had passed. We also sought to look at the effect of DHS campaigns on this population’s understanding of the pandemic and any change in behaviour as a result of the campaign.

## Methods

The cross-sectional study took place in the ED of the Royal Melbourne Hospital (RMH), Australia. RMH is a university affiliated tertiary referral hospital in Melbourne, Victoria. Approximately 58,000 patients attend the ED per annum, with an admission rate of approximately 40%.

The survey instrument was based on similar published surveys conducted on this subject, Severe Acute Respiratory Syndrome (SARS) and avian influenza [6-10]. It was assessed by a panel of physicians and piloted in the RMH ED. The final survey, which contained 42 questions, was in written format, only available in English and took five to ten minutes to be completed (see Additional file 1). The study protocol was approved by the Melbourne Health Research Ethics Committee.

The questionnaire was administered to a convenience sample of patients, visitors, non-clinical staff and volunteers of the RMH ED and names were not recorded. Eligible respondents were 18 years or older, had sufficient English proficiency and did not present with an influenza-like illness. The survey was administered by one researcher (NJ) between the hours of 0900 and 2100 on all days of the week from the 15th of February to the 22nd of March 2010. The sample size of 252 respondents was a convenience sample of patients in the ED over the study period who were available to be approached by the investigators. A target of 250 patients was chosen to provide a probable broad representation of the ED population.

The survey instrument consisted of questions about the following:

1. Source of information about pandemic (H1N1) 2009 influenza and recall of DHS communications

2. Knowledge assessment of pandemic (H1N1) 2009 influenza (symptoms, mode of transmission, incubation period, vulnerable groups, precautionary measures)

3. Perceived personal risk

4. Perceptions about government and media coverage of the pandemic situation in Australia (Likert-type scale of strongly agree to strongly disagree)

5. Likely compliance with future pandemic measures

6. Personal and household demographics

A knowledge score was constructed from responses to questions about symptoms, mode of transmission, incubation period, precautions and vaccination. One mark was attributed to each correct answer to these questions (total of 28 marks).

**Figure 1:**
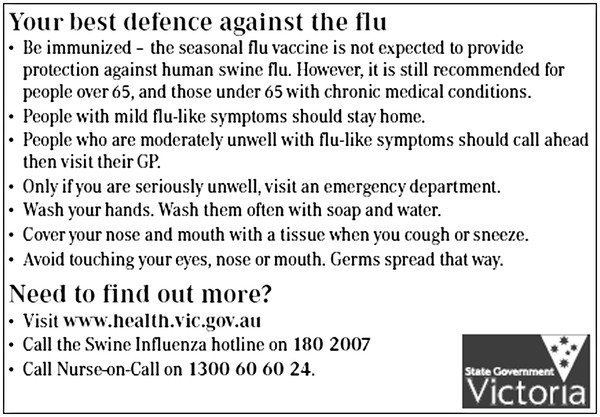
Pandemic (H1N1) 2009.

Public education campaigns conducted on pandemic (H1N1) 2009 influenza were used to ascertain the knowledge of respondents. An example of an advertisement placed in newspapers is shown in Figure 1 (Personal communication from Lester R. to CM 2009).

The pandemic (H1N1) 2009 influenza vaccine became available on the 30th of September 2009 [11]. Public health campaigns stated that the 2009 seasonal flu vaccine would not provide protection against the pandemic strain but still recommended the seasonal vaccine for vulnerable groups (Personal communication from Lester R. to CM 2009). The 2010 seasonal vaccine which incorporates the pandemic (H1N1) 2009 influenza vaccine became available after our survey.

Responses were entered in an Excel® database and presented descriptively with 95% confidence intervals and summary statistics where appropriate. Knowledge score for those who recalled the DHS campaign was compared to those who did not recall it using the Wilcoxon Sign-Rank test on Stata Version 10 (College Station Tx).

## Results

### Demographic characteristics

A total of 317 potential participants were approached. Of these, 21 (6.6%) declined to participate and 14 (4.4%) were excluded because of insufficient English. Of the 252 who were recruited, seven (2.8%) went in for consultation or treatment before finishing the questionnaire but were included in the final sample. The median age was 36 (range 18 to 83) years. Table 1 shows the demographic characteristics of the population to whom the questionnaire was administered.

**Table 1:** Demographic characteristics of respondents (n = 252)

**Characteristic**	**Frequency**	**%**	**95% CI**
Female	121	48.0	41.7–54.4
Males	132	52.0	45.8–58.2
**Place of birth**			
Australia	150	59.5	53.1–65.6
Other countries^1^	88	34.9	29.1–41.2
**Language spoken at home**			
English only	206	81.7	76.3–86.2
Language other than English	31	12.3	8.6–17.2
English and another main language ^2^	7	2.8	1.2–5.9
Did not answer	8	3.2	1.5–6.4
**Highest Educational attainment**			
None	1	0.4	0.0–2.5
Primary	3	1.2	0.3–3.7
Secondary	94	37.7	31.8–44.0
Tertiary	134	55.9	49.5–62.1
Did not answer	12	4.8	2.6–8.4
**Living arrangements**^**3**^			
Partner	124	49.2	42.9–55.5
Children	71	28.2	22.8–34.3
Other family	40	15.9	11.7–21.1
Friends	39	15.5	11.3–20.7
Live alone	34	13.5	9.7–18.5
Did not answer	11	4.4	2.3–8.0
**Having school aged children**			
Yes	62	28.2	22.8–34.3
No	180	67.4	61.2–73.1
Did not answer	10	4.4	2.3–7.9
**Employment status**			
Full time	111	44.0	37.8–50.4
Student	41	16.3	12.1–21.6
Part time	36	14.3	10.3–19.4
Retired	28	11.1	7.6–15.8
Casual	26	10.3	7.0–14.9
Other ^4^	12	4.8	2.6–8.4
Unemployed	9	3.6	1.8–7.0
**Can work from home**^**5**^			
Yes	46	18.3	13.8–23.8
No	131	52.0	45.7–58.3
Do not work	55	21.8	17.0–27.5
Did not answer	20	7.9	5.0–12.1
**Internet Use**			
At least once a week	180	71.4	65.3–76.8
Less often than once per week	61	24.2	19.1–30.1
Did not answer	11	4.4	2.1–7.6
**Status (N = 133)**			
Patient	65	48.9	42.6–55.2
Visitor	55	41.4	35.3–47.8
Volunteer	3	2.3	1.0–5.3
Non-clinical hospital staff	5	3.8	1.9–7.2
Tourist ^6^	5	3.8	1.9–7.2
**Contracted pandemic (H1N1) 2009 Influenza**			
Throat swab confirmed	2	0.8	0.1–3.2
Doctor confirmed on clinical basis	9	3.6	1.8–7.0
Participant thought so based on symptoms	10	4.0	2.1–7.4
No	211	83.7	78.4–87.9
Unsure or did not answer	20	7.9	5.0–12.1

^1^Other main countries: United Kingdom, India, China.

^2^Other languages – Chinese, Pilipino, Greek, Maltese, Lebanese.

^3^Adds to greater than 100% as participants were allowed to answer yes to more than one question.

^4^ Pensioner, self employed.

^5^Willing to work from home as a percentage of those employed = 23.4%.

^6^On a short stay visit to Australia: were not present in Australia during the pandemic period in 2009, nor will be present for the winter season in Australia in 2010.

**Table 2:** Perceptions about pandemic (H1N1) 2009 influenza severity and progression (n = 252)

	**Frequency**	**%**	**95% CI**
**H1N1 is a very serious disease**			
Strongly agree	59	23.4	18.4–29.2
Agree	107	42.3	36.2–48.7
Neutral	52	20.6	15.9–26.2
Disagree	21	8.3	5.3–12.6
Strongly disagree	6	2.8	1.2–5.9
**Most people who catch pandemic (H1N1) 2009 influenza die**			
Strongly agree	9	3.6	1.8–6.9
Agree	19	7.6	4.8–11.8
Neutral	41	16.3	12.1–21.6
Disagree	126	50.0	43.7–56.3
Strongly disagree	51	20.2	15.5–25.8
**Pandemic (H1N1) 2009 influenza has ended in Australia**			
Strongly agree	9	3.6	1.8–6.9
Agree	45	17.9	13.5–23.3
Neutral	90	35.7	29.9–42.0
Disagree	78	31.0	25.4–37.2
Strongly disagree	21	8.3	5.3–12.6
**Where is one most likely to catch pandemic (H1N1) 2009 influenza?**^**1**^			
Public transport	178	70.6	64.5–76.1
Caring for somebody sick with pandemic (H1N1) 2009 influenza	169	67.1	60.9–72.8
Workplace	161	63.9	57.6–69.8
Hospital	122	48.4	42.1–54.7
Indoor sports events	104	41.3	35.2–47.7

^1^ allowed to answer more than once.

### Source of information

**Figure 2:**
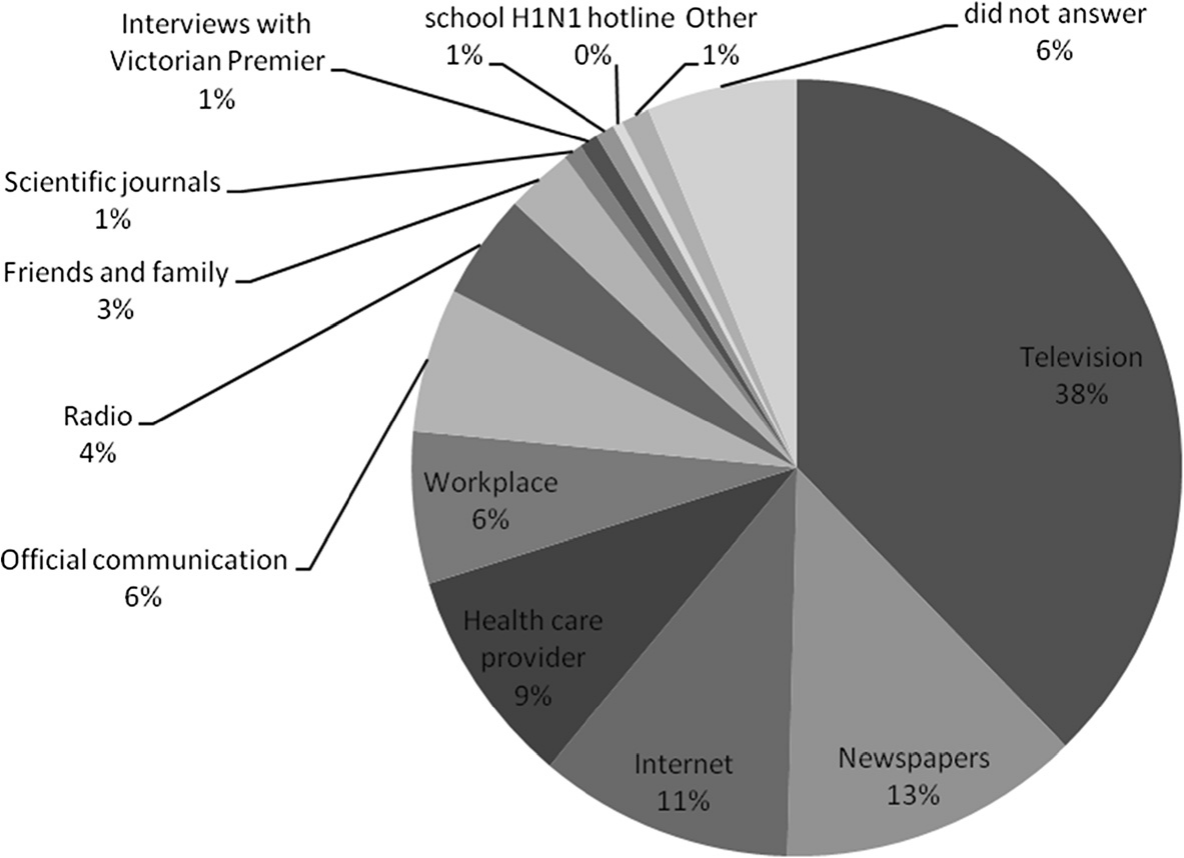
Source of information about pandemic (H1N1) 2009 influenza.

Figure 2 shows the different sources of information used by participants. Over half of the participants obtained information from television or newspapers (both traditional forms of mass media). Information obtained from the internet was mainly composed of general websites and online newspapers. Twitter updates used for the first time by the DHS were uncommonly accessed (3 [2.7%]). More than half of respondents (144 [57%]) were aware of communications from the DHS.

### Knowledge and misconceptions about pandemic (H1N1) 2009 influenza

#### Symptoms

More than half of respondents correctly identified the main features of pandemic (H1N1) 2009 influenza infection (fever 224 [92%], body aches 179 [77%], headaches 177 [76%], cough 166 [72%], chills 113 [52%]) although only 19% (35) correctly identified diarrhoea as a possible symptom.

#### Transmission

Person-to-person transmission was recognised by the majority of respondents (215 [85%]) as a mode of transmission. However, only 37% (94) of respondents identified that contact with contaminated objects could be a source of infection. Almost a quarter of respondents (60 [24%]) thought that live pigs could transmit the infection.

#### High risk groups

The elderly (185 [73%]), pregnant (181 [72%]) and children (165 [66%]) were correctly identified as being at high risk whereas the obese (45 [18%]) and the indigenous groups (30 [12%]) were not as well recognised as being vulnerable groups.

#### Precautions

**Figure 3:**
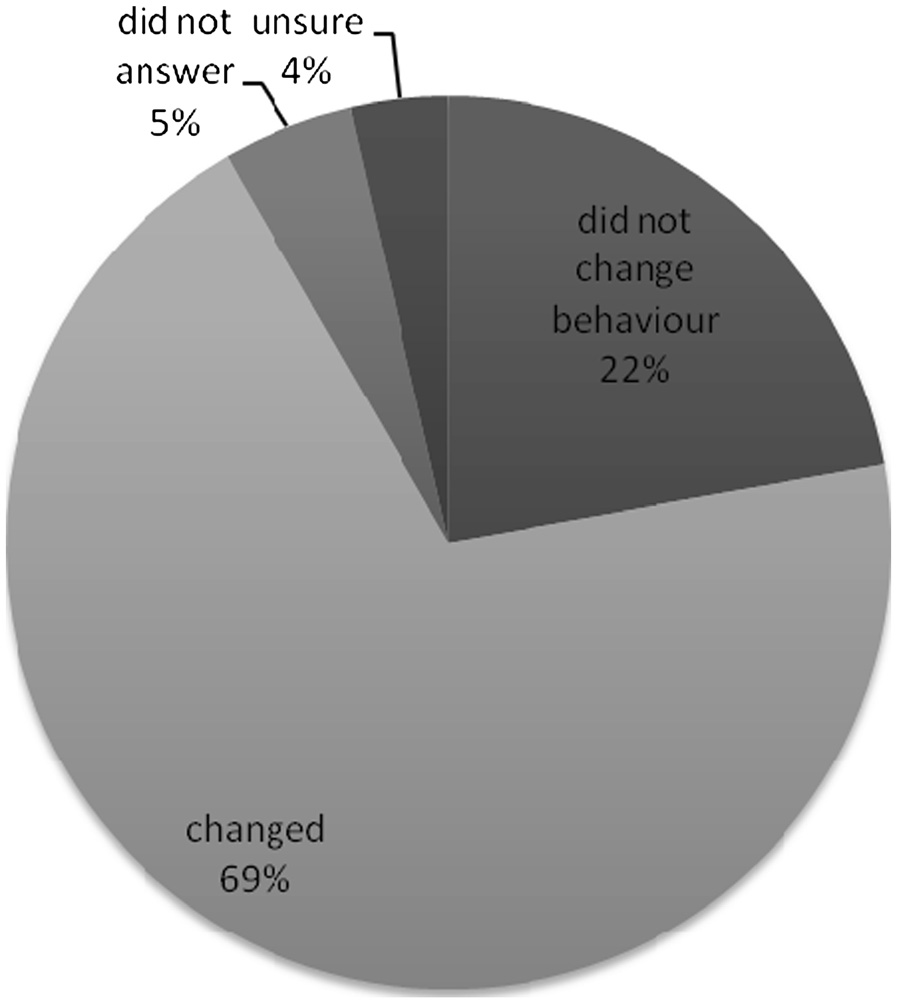
Behaviour change in response to pandemic (H1N1) 2009 influenza.

The three main messages conveyed by the DHS (hand washing with soap and water, covering nose and mouth while coughing and sneezing and throwing tissues in the rubbish bin after using them) were correctly identified by over three-quarters of the population. However, only 26% (66) and 29% (74) of respondents respectively recalled two other DHS campaign messages that they should see their general practitioner if moderately unwell or the ED if seriously unwell. More than half of the respondents (136 [54%]) reported they would wear facemasks despite this not being a DHS recommendation for the public. Figure 3 shows how the participants reported changing their behaviour in response to the pandemic.

Intended compliance with future pandemic measures such as working from home (109 [43.3%]), postponing social gatherings (88 [34.9%]) or wearing facemasks (95 [37.7%]) was low. Mandatory oseltamivir treatment if sick was more acceptable (158 [62.7%]).

#### Knowledge score

**Figure 4:**
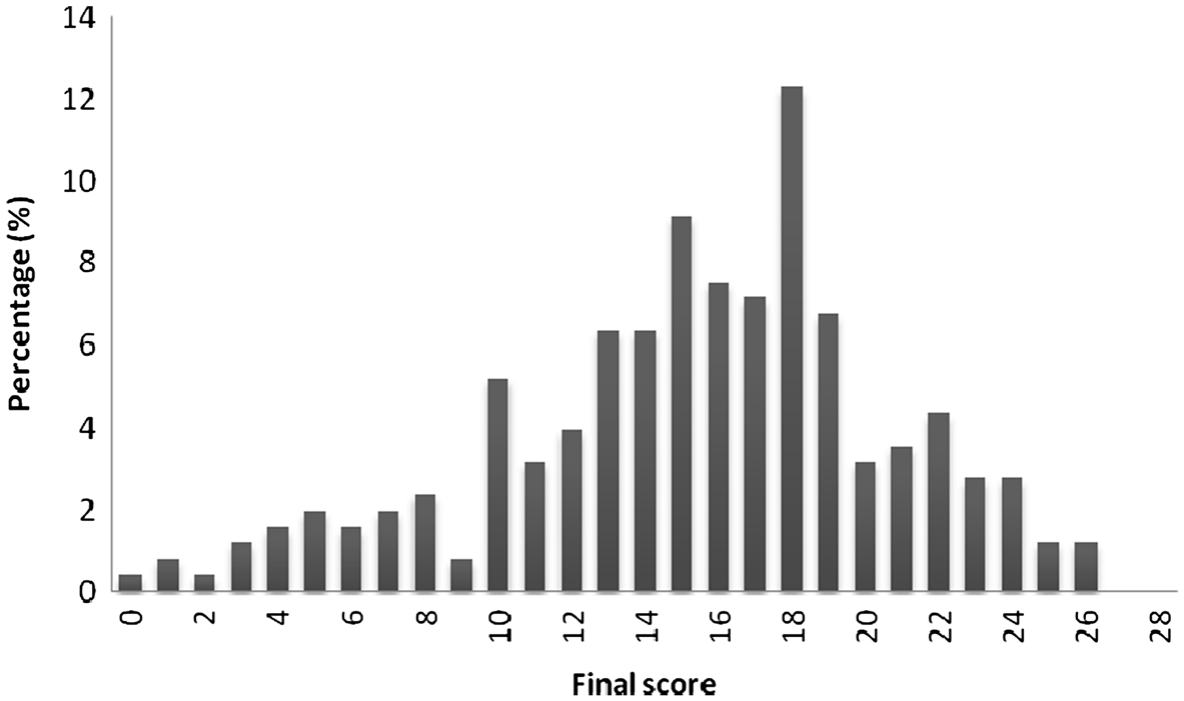
Knowledge score distribution (N = 200, maximum = 28).

Figure 4 shows the distribution of participant knowledge scores. Those who recalled the DHS campaign had a significantly higher median knowledge score than those who did not (17/28 versus 14/28 – 28 being the highest mark on the knowledge score) (p = 0.0008).

#### Perceptions about pandemic (H1N1) 2009 influenza and vaccination

Table 2 shows the respondents beliefs about pandemic (H1N1) 2009 influenza. Seventy per cent of respondents did not consider pandemic (H1N1) 2009 influenza to be potentially fatal but still considered it to be a serious disease. 43% of the population interviewed did not think that the pandemic was likely to recur compared to only 21% who thought that the pandemic would return. Table 3 shows the respondents attitudes towards influenza vaccination.

**Table 3:** Attitudes towards vaccination (n = 252 unless stated otherwise)

	**Frequency**	**%**	**95% CI**
**Seasonal influenza vaccine 2009**			
Does not protect against pandemic (H1N1) 2009 influenza	138	54.8	48.4–61.0
Unsure	60	23.8	18.8–29.6
Never had seasonal vaccine	109	43.3	37.1–49.7
**Pandemic (H1N1) 2009 influenza vaccine**			
Had the new vaccine	56	22.2	17.3–27.9
Intend to have it	36	14.3	10.3–19.4
Neither	154	61.1	54.8–67.1
**Reasons for not getting the new vaccine or not intending to get it (n = 154)**			
Low risk patient	47	30.5	25.0–36.7
Vaccine has side effects	30	19.5	14.9–25.1
Could not be bothered	27	17.5	13.1–22.9
The new pandemic (H1N1) 2009 influenza vaccine may not be effective next year due to viral changes	24	15.6	11.5–20.8
Prepared to wait for winter 2010	18	11.7	8.1–16.5

#### Perceptions about the government and media

Table 4 shows attitudes of respondents about the local authorities and the media. Most respondents (108 [42.8%]) agreed that the media gave them a good idea of what to expect during the pandemic. A similar proportion (107 [42.4%]) also thought that too much repetitive information from the media had lead to them to lose interest in the pandemic.

**Table 4:** Perceptions of government and media during pandemic (H1N1) 2009 influenza (n = 252)

	**Government (%)**			**Media (%)**		
	**Frequency**	**%**	**95% CI**	**Frequency**	**%**	**95% CI**
**Information provided during the pandemic**						
Very clear and specific or somewhat clear and specific	112	44.4	38.2–50.8	112	44.4	38.2–50.8
Neutral	61	24.2	19.1–30.1	60	23.9	18.9–29.7
Somewhat unclear and confusing or very unclear and confusing	59	23.4	18.4–29.2	60	23.9	18.9–29.7
**Threat communication about the pandemic**						
Over-exaggerated or mildly exaggerated the threat	100	39.7	33.7–46.1	146	57.9	51.5–64.0
Neutral	103	40.9	34.8–47.3	72	28.6	23.2–34.7
Mildly downgraded or overall downgraded the seriousness of the situation	28	11.1	7.6–15.8	13	5.2	2.9–8.9

## Discussion

This is the first Australian study to correlate the general public’s knowledge of pandemic (H1N1) 2009 influenza with a health department public health campaign. It identified that this ED population had a good understanding of the clinical features of pandemic influenza and of the precautions required to minimise its spread in the community. Health literacy and behavior change were reported much more frequently than in similar studies conducted in 2009 and demonstrate that this ED population potentially has the capacity to respond effectively to an outbreak [12-14]. For comparison, Kamate in India and Rubin in London both showed only a 40% to 50% change in behavior for this pandemic period whereas our study showed that up to 69% of respondents had had some form of behavior change [12,13]. Our results also showed a significantly higher knowledge score for respondents who specifically remembered the DHS campaign. This suggests the importance of pandemic communications in increasing the health literacy of hospital-based populations.

Person-to-person transmission of the virus was recognised by 85% of people. However, the early use of the moniker “swine flu” and the launching of the Australian campaign “The Facts about Swine Flu” may have contributed to more than 25% of respondents thinking that pigs could transmit the infection [15]. This could impede measures aimed at reducing person-to-person transmission by introducing ambiguity.

Seventy per cent correctly identified pregnant women as a vulnerable group, a central DHS message at the onset of the outbreak and during vaccination campaigns suggesting the effectiveness of the DHS in publicising key information. Indigenous groups were not recognized as being vulnerable by 85% of respondents. This could be explained by this study being conducted in Victoria, which has a smaller Indigenous population compared to other states [16].

The three main precautionary messages publicised by the DHS were identified by 80% of respondents and this probably shows that the messages were well emphasised. However, 70% of respondents did not know that visiting the GP or the ED was only recommended if one’s condition deteriorated. However, as this message changed as the pandemic progressed – early in the pandemic all symptomatic persons were encouraged to present to a medical service – this is not surprising. As explained in Elledge’s pandemic planning study, measures aimed at protecting others (here vulnerable patients at the GP) are not implemented as rigorously as measures to protect the individual [17].

Studies conducted by Kamate, Lau and Goodwin showed that 25% to 40% of their participants had the misconception that the current seasonal vaccine would be effective against the pandemic strain [7,14,18]. This is reflected in that almost half of our respondents thought that seasonal influenza vaccine 2009 would be effective against the new viral strain. This may have resulted from concurrent campaigns in 2009 still recommending “getting the seasonal vaccine”.

This study also showed that despite 80% knowing about the new vaccine, only 20% had received it. This is consistent with Kiviniemi’s study on the willingness to be vaccinated where only 16% of the population would want to be vaccinated, in contrast to the 98% willing to adopt preventative measures such as hand washing [19]. The Protection Motivation Theory postulates that perceived vulnerability increases compliance with vaccination and precautionary measures [20]. In our study, perception of being at low risk proved a greater deterrent than potential side-effects. This is in accordance with the above theory and Van Der Weerd’s study in the Netherlands, where willingness to be vaccinated increased six-fold between the time that the WHO announced a pandemic alert level of phase five to the infection causing its first deaths in the country [20]. Vaccination could be increased by campaigns emphasising how low risk groups can still transmit the infection to more vulnerable family members, even if only mildly unwell themselves, and that severe influenza can still affect groups perceived to be at low risk of severe disease.

Another finding was that use of traditional mass media was prevalent whereas new technology such as Twitter updates was not. The use of the internet, mainly composed of viewing online newspapers, was higher than other pandemic studies. For example, Kim’s study on SARS in 2003 showed only three percent of the population had access to the internet to find out about the disease [6]. It is surprising that higher results were not obtained for newer forms of communication since this sample was composed of relatively young people with a median age of 36. Thus, it would appear that future campaigns should still emphasize traditional modes of communication.

This study found that the government and media were thought to have provided sufficient information but that the message was repetitive and led to loss of interest in the issue. “Pandemic fatigue” is of concern because it may lead to lower compliance with public health measures as evidenced in focus group studies conducted by Elledge and Rogers where participants warned against tiresome public health announcements [17,21]. Moreover, participants thought that both the government and the media had exaggerated the threat posed by the pandemic. This is in contrast to Fogarty’s risk communication study which concluded that television reporting of pandemic (H1N1) 2009 pandemic was not generally alarmist [22]. This might have led to decreased risk perception, which as demonstrated in Lau’s study in Hong Kong during the SARS epidemic, has been shown to undermine the credibility of authorities [23].

Waterer also reported that mistakenly believing WHO pandemic phases to be a measure of the severity may have led to thinking it was an exaggeration and may explain the attitude of our participants to government and media on the matter [24]. If there is a more severe influenza pandemic in the future, communications should address the feeling of exaggeration and the loss of interest to ensure prompt compliance with pandemic measures.

This study also found that only 40% of respondents intended to adhere to government recommendations in case of a future pandemic and is lower than reported in other pandemic planning studies. After the relatively mild impact of pandemic influenza, this ED population did not believe that future pandemics would be serious enough to mandate preventative measures. Campaigns should enforce the idea that future pandemics may be more severe than the recent one.

This study was restricted to a population consisting of ED attendees and was limited to English-speaking participants who were well enough to participate. This may result in the study not being generalisable to other populations. However, there was a range of educational and economic backgrounds in our sample. Further research is required to determine the effect of DHS campaigns on more culturally and linguistically diverse groups. Although this was a convenience sample and sample size was not computed *a priori*, this was meant to be a hypothesis generating observational study and we have presented 95% confidence intervals, which are generally quite narrow, indicating adequate sample size.

This survey also posed hypothetical questions and even though intentions have been shown to predict behaviour, the answers may not reflect real-life responses [25]. Moreover, the attitudes and knowledge of our subjects are not static and may evolve over time depending on a number of factors including the severity of influenza in a given season. This study was conducted over only a few months in the aftermath of the pandemic and the public health effects demonstrated by this study is liable to change with time. A subsequent study may be needed to determine this. Nevertheless, the short term outcome demonstrated is relevant since the public health campaign targets immediate rather than belated behavioral changes in response to a pandemic threat.

## Conclusions

This study shows that this ED population has the capacity to respond effectively to pandemic measures and that appropriate health literacy was achieved. The knowledge score was significantly higher for those participants who remembered DHS messages, demonstrating their importance in health literacy. Future campaigns should build on this result and address the misconceptions about mode of transmission, high risk groups and precautionary measures. Compliance with vaccination needs to be addressed because it has direct implications for the annual influenza season. The study showed that traditional mass media was an important mode of pandemic communication even for the younger population.

## Competing interests

The authors declare that they have no competing interests.

## Authors’ contributions

The study was conceived by JK, CM and NJM. Data collection was conducted by NJM. All authors read and approved the final manuscript.

## Supplementary Material

Additional file 1 **Survey instrument. **This file contains the 10 page questionnaire administered to participants. Key sections have been reported in this paper. Click here for file
