# Supplementary material for: A cross-sectional study of pandemic influenza health literacy and the effect of a public health campaign
Source: BMC Res Notes. 2012 Jul 26;5:377. doi: 10.1186/1756-0500-5-377 (PMC3502135; doi:10.1186/1756-0500-5-377)
Supplement: Additional file 1 — Survey instrument. This file contains the 10 page questionnaire administered to participants. Key sections have been reported in this paper. [file 1756-0500-5-377-S1.doc]

# Health literacy of H1N1 2009 Influenza amongst patients, visitors and non-clinical staff of an Australasian Emergency Department

The information collected for this study is anonymous and will be used to determine how well Some Emergency Department staff, visitors and patients understand swine flu and where they gained their knowledge. Participation is entirely voluntary.

# H1N1 2009 (swine origin influenza) Survey

## Source of information

1. **Where did you get information on the recent swine flu pandemic?**

**Tick all that apply**

- Press conferences and interviews with the Premier, Health minister or Chief Medical Officer
- Official communication (pamphlets, posters, factsheets and advertisement) from the Department of Health (Department of Human Services)
- Swine Influenza telephone hotline
- Your workplace (if applicable)
- University/school announcements
- Hospital or Health practitioner e.g. General practitioner, Dentist, physiotherapist, chiropractor, pharmacist, nurse
- Friends and Family
- Television
- Internet
- Radio
- Newspapers
- Scientific journals
- Other __________________________ (please specify)
- Unsure

1. **Which of the above do you think was the most useful source of information for you?**

_________________________

1. **If you used the internet as a source of information, where did you find that information on the internet? Tick all that apply**

- General search for swine flu on Google, Yahoo etc
- Online newspapers or internet news channels on msn/yahoo etc.
- Department of health pandemic website
- Twitter feeds from the Department of Health
- Center for Disease Control (US) website or other official web site eg WHO
- Other: __________________ (please specify)
- Unsure

1. **Do you remember seeing the posters and advertisements from the Department of Health on TV, radio and in newspapers/magazines?**

Yes□ No□ Not sure□

## Knowledge of swine flu

1. **To your knowledge, which of the following are symptoms of swine flu?**

**Tick all that apply**

- Fever
- Itch
- Chills
- Double vision
- Headache
- Body aches
- Diarrhoea
- Constipation
- Pain on urination
- Cough
- Unsure

1. **How is swine flu transmitted? Tick all that apply**

- Insect bites
- From live pigs to people
- From birds to people
- From eating cooked pork
- From eating badly cooked chicken
- Through the ventilation or the air conditioning system
- From person to person through sneezing, coughing, talking
- Touching an infected person
- By touching contaminated objects like doorknobs or toys
- Drinking tap water
- Other ______________________________ (please specify)
- Unsure

1. **Where is one very likely to catch swine flu? Tick all that apply**

- At home caring for somebody who has swine flu
- In the workplace
- Visiting a hospital
- Walking down the street
- On public transport
- Major outdoor sporting events
- Major indoor sporting events
- University or other learning institution
- Other: ______________________ (please specify)
- Unsure

1. **Once you have been infected with swine flu, how long does it take to start getting symptoms?**

- Less than one week
- One to two weeks
- Greater than two weeks
- Unsure

1. **Which of the following groups are at risk of more severe complications from swine flu? Tick all that apply**

- Pregnant women
- Diabetics
- Adolescents
- Children younger than 2 years old
- Elderly (greater than 65 years old)
- Vegetarians
- Asthmatics
- The obese
- IV drug users
- Indigenous Australians
- Urban dwellers (people who live in the city)
- Other: ____________________________(please specify)
- Unsure

1. **Which measures SHOULD be taken to protect YOU from getting swine flu? Tick all that apply**

- Hand-washing with soap and water
- Cleaning the house more often
- Keeping the children from school
- Wearing warmer clothes than usual
- Avoiding touching eyes, nose and mouth
- Eating a balanced diet and exercising regularly
- Getting the new swine flu vaccine
- Getting the seasonal flu vaccine
- Taking Tamiflu
- Avoid crowded places
- Avoid going overseas
- Other: __________________________________(please specify)
- Unsure

1. **Which of the following can slow the spread of swine flu IN THE COMMUNITY?**

**Tick all that apply**

- Hand-washing with soap and water
- Covering nose and mouth with a tissue when sneezing or coughing
- Throwing your tissues in the bin after using them
- School closures
- Cancelling major events (e.g. sports competitions, festivals, concerts etc)
- Using facemasks
- Getting the new swine flu vaccine
- Staying at home if mildly unwell so as not to infect other members of the community
- Only go to the GP if moderately unwell so as not to infect other people at the GP practice
- Only go to the emergency department if seriously ill so as not to infect other people at the hospital
- Avoiding crowded places so as not to infect other members of the community
- Unsure
- Other: ________________________(please specify)

1. **How did you change your behaviour during the pandemic?**

- I increased my frequency of hand washing
- I was more cautious while sneezing and coughing, e.g. paying more attention to covering my mouth, using tissues and throwing them away in a bin
- I used commercially available alcohol-based hand sanitisers
- I avoided overseas travel
- I avoided going to major social events such as sporting matches, festivals or parties
- I avoided sending my children to school even if the school was NOT closed
- I avoided public transport
- I avoided going to work
- I avoided mingling with other people who were sick
- I wore face masks
- I kept a stock of Tamiflu
- I got the seasonal flu vaccine
- I adopted healthy habits such as exercising more regularly and eating a balanced diet
- I wore warmer clothes to stop from catching swine flu
- Other ________________________(please specify)
- Unsure
- I did not change my behavior

1. **Please state whether the following are TRUE or FALSE:**

The seasonal influenza vaccine can prevent swine flu

True □ False □ don’t know □

Cold and flu tablets from the pharmacy can cure swine flu

True □ False □ don’t know □

1. **Do you usually get seasonal influenza vaccine?**

Yes, in the past□ Yes, in 2009□ Never□

1. **Have you had the new swine flu vaccine?**

Yes □ No□

1. **If no, do you intend to get it in the next few weeks?**

Yes □ No□

1. **If No to both 15 and 16, why not?**

- Will wait until next winter when the seasonal vaccine is available
- The vaccine may have serious side effects
- Swine flu is usually mild
- I am not in a high risk group for severe disease
- I have already had swine flu
- The new swine flu vaccine may not be effective next year due to viral changes
- The vaccine from the last influenza season is enough
- Cannot afford it
- Cannot be bothered
- Other ____________________ _____(please specify)

## Perceptions about swine flu

1. **I believe that swine flu is a very serious disease.**

| Strongly agree□ | Agree □ | Neutral□ | Disagree□ | Strongly disagree □ |
| --- | --- | --- | --- | --- |

1. **Most people who catch swine flu die.**

| Strongly agree□ | Agree □ | Neutral□ | Disagree□ | Strongly disagree □ |
| --- | --- | --- | --- | --- |

1. **I believe that the swine flu epidemic has ended in Australia:**

| Very confident□ | Somewhat confident □ | Neutral□ | Not very confident □ | Not at all confident □ |
| --- | --- | --- | --- | --- |

1. **Did you catch swine flu?**

- Yes and I had a throat swab that confirmed that I had swine flu
- Yes because the doctor informed me that I might have swine flu but I did NOT get a throat swab
- Yes : I think it is very likely that I caught swine flu but I did NOT go to the doctor
- No, I did not get swine flu
- Unsure

## Government and media

**How did the Government/Department of Health/ Health minister/ Chief Health Officer handle this pandemic?**

1. They had a well prepared influenza pandemic plan

| Strongly agree□ | Agree □ | Neutral□ | Disagree□ | Strongly disagree □ |
| --- | --- | --- | --- | --- |

1. How was the information provided by the government about what to do during a pandemic?

| very clear and specific□ | somewhat clear and specific □ | Neutral□ | somewhat unclear and confusing□ | very unclear and confusing □ |
| --- | --- | --- | --- | --- |

1. How did the government communicate about the threat posed by swine flu?

| Over-exaggerated the threat□ | Mildly exaggerated the threat□ | neutral□ | Mildly downgraded the seriousness of the situation□ | Overall, downgraded the seriousness of the situation□ |
| --- | --- | --- | --- | --- |

***If you did not watch the media (television, radio, news, newspapers, magazines), tick □ and move to next question***

**How did the media (television and radio news, newspapers, magazines) handle the pandemic?**

1. They gave a good idea of what to expect during the pandemic

| Strongly agree□ | Agree □ | Neutral□ | Disagree□ | Strongly disagree □ |
| --- | --- | --- | --- | --- |

1. Too much repetitive information eventually made me lose interest in swine flu

| Strongly agree□ | Agree □ | Neutral□ | Disagree□ | Strongly disagree □ |
| --- | --- | --- | --- | --- |

1. **How did the MEDIA communicate about the threat posed by swine flu?**

| Over-exaggerated the threat□ | Mildly exaggerated the threat□ | neutral□ | Mildly downgraded the seriousness of the situation□ | Overall, downgraded the seriousness of the situation□ |
| --- | --- | --- | --- | --- |

1. How was the information provided from the media about the pandemic?

| Very clear and with a lot of useful detail□ | Somewhat clear and with details□ | Neutral & balanced□ | Somewhat unclear and inconsistent□ | Very unclear and inconsistent□ |
| --- | --- | --- | --- | --- |

1. **Would you comply with the following governmental measures if the pandemic reappears?**
   1. Work from home if possible → Yes□ No□ Unsure□
   2. Postpone social gatherings → Yes□ No□ Unsure□
   3. Purchase face masks and wear them → Yes□ No□ Unsure□
   4. Take antivirals such as Tamiflu if you are sick→ Yes□ No□ Unsure□

## Respondent Characteristics

1. **Gender**: Female □ Male□
2. **How old are you?** ___________ years old
3. **Where were you born?** _________________________
4. **Do you speak mainly English at home?** Yes □ No□
5. **If No, what do you mainly speak?** _______________________________
6. **What is your highest education attainment?**

- None
- Primary School
- Secondary school
- Tertiary

1. **Postcode: _ _ _ _**
2. **Living arrangements – Tick if more than one**

- with partner
- with children
- with other family
- with friends
- live alone

1. **Do you have school age children?**

Yes □ No □

1. **Employment status**

- Employed

□Part-time □ Full-time □ Casual

- Home duties
- Unemployed
- Student
- Retired
- Other

1. **If you are employed, can you work from home if the need arises?**

Yes □ No□

1. **If you are employed, what is your average annual personal income ($)**

- <20 000
- 20 000 to 55 000
- >55 000

1. **Do you regularly use the internet (once a week or more)?**

Yes □ No□

***

## Thank you for kindly giving your time to participate in this study
